# Arsenic Inhibits Neurite Outgrowth by Inhibiting the LKB1–AMPK Signaling Pathway

**DOI:** 10.1289/ehp.0901510

**Published:** 2009-12-22

**Authors:** Xin Wang, Dan Meng, Qingshan Chang, Jingju Pan, Zhuo Zhang, Gang Chen, Zunji Ke, Jia Luo, Xianglin Shi

**Affiliations:** 1 Graduate Center for Toxicology, University of Kentucky, Lexington, Kentucky, USA; 2 Key Laboratory of Nutrition and Metabolism Institute, Institute for Nutritional Sciences, Shanghai Institutes for Biological Sciences, Chinese Academy of Sciences, Shanghai, People’s Republic of China; 3 Department of Internal Medicine, University of Kentucky, Lexington, Kentucky, USA

**Keywords:** AMPK, arsenic, developmental neurotoxicity, LKB1, neurite outgrowth, neuro-2a neuroblastoma cell, ROS

## Abstract

**Background:**

Arsenic (As) is an environmental pollutant that induces numerous pathological effects, including neurodevelopmental disorders.

**Objectives and Methods:**

We evaluated the role of the LKB1–AMPK pathway in As-induced developmental neurotoxicity using Neuro-2a (N2a) neuroblastoma cells as a model of developing neurons.

**Results:**

The addition of low concentrations of As (≤ 5 μM) during differentiation caused an inhibitory effect on the neurite outgrowth in N2a cells in the absence of cell death. Activation of adenosine monophosphate–activated kinase (AMPK) induced by retinoic acid in differentiating cells was blocked by As. Pretreatment with the AMPK-specific activator 5-aminoimidazole-4-carboxamide riboside or overexpression of a constitutively active AMPK-α1 plasmid reversed As-induced inhibition of neurite outgrowth. The activation of LKB1 (serine/threonine kinase 11), a major AMPK kinase, was also suppressed by As by inhibiting both the phosphorylation and the translocation of LKB1 from nucleus to cytoplasm. Antioxidants, such as N-acetyl cysteine and superoxide dismutase, but not catalase, protected against As-induced inactivation of the LKB1–AMPK pathway and reversed the inhibitory effect of As on neurite outgrowth.

**Conclusions:**

Reduced neurite outgrowth induced by As results from deficient activation of AMPK as a consequence of a lack of activation of LKB1. Oxidative stress induced by As, especially excessive superoxide, plays a critical role in blocking the LKB1–AMPK pathway. Our studies provide insight into the mechanisms underlying As-induced developmental neurotoxicity, which is important for designing a new strategy for protecting children against this neurotoxic substance.

Underground water in many regions of the world is contaminated with arsenic (As), and the resulting toxicity has created a major environmental and public health problem in the affected regions. Chronic As exposure can cause many diseases, including neurodevelopmental disorders and brain disease. Arsenic—along with As compounds—is one of the five industrial chemicals proven to cause neurodevelopmental disorders among thousands of known chemicals (Grandjean and Landrigan 2006). In rats, As exposure during the rapid brain-growth period causes faulty migration, delayed maturation, and alteration in nuclear area measurements of Purkinje cells in the cerebellum and impaired brain structural organization and the shape of fiber tracts and axons in the striatum (Dhar et al. 2007; Rŕos et al. 2009). Although accumulating evidence for neurodevelopmental neurotoxicity of As is established, the causative mechanism remains unclear. The toxic effects of As on children have generally been overlooked, and regulatory action does not emphasize the need to protect the developing brain against this neurotoxic substance (Mukherjee et al. 2006).

Adenosine monophosphate-activated kinase (AMPK) is an important integrator of signals that control cellular energy balance through the regulation of multiple biochemical pathways (Hardie et al. 1999). Recent studies have suggested that AMPK also regulates cell structure and polarity, cell division, and normal growth and development (Dasgupta and Milbrandt 2009; Lee et al. 2007). AMPK helps maintain genomic integrity in neuron precursors and the structure and function of mature neurons in *Drosophila* (Lee et al. 2007). Loss of AMPK activity causes neurodegeneration in *Drosophila* (Spasić et al. 2008) and structural and functional brain abnormalities in *AMPK*-mutant mice (Dasgupta and Milbrandt 2009). The activation of AMPK contributes to the stimulating effect of resveratrol on neurite outgrowth in neurons (Dasgupta and Milbrandt 2007). These studies suggest that AMPK may have additional roles beyond the established metabolic functions, both in neuronal development and in neurodegenerational diseases.

AMPK exists in cells as a heterotrimeric complex containing a catalytic α subunit, a scaffolding β subunit, and an AMP-binding γ subunit. The activation of AMPK requires phosphorylation of Thr172 in the activation loop of the catalytic α subunit [AMPKα(Thr172)] (Shaw et al. 2004). Recent studies have identified LKB1 (serine/threonine kinase 11) and Ca^2+^/calmodulin-dependent protein kinase kinase-β (CaMKKβ) as two kinases that phosphorylate Thr172 (Carling et al. 2008; Woods et al. 2003). LKB1 signaling is regulated through two main mechanisms: phosphorylation and subcellular localization. In resting cells, LKB1 is reported to be predominantly located in the nucleus (Tiainen et al. 1999); however, the cytoplasmic localization of LKB1 is critical for its normal function (Boudeau et al. 2003). LKB1 forms a heterotrimeric complex with the regulatory proteins ste20-related adaptor (STRAD) and mouse protein 25 (MO25), which causes a relocalization of LKB1 to the cytosol and enhances LKB1 activity (Shaw et al. 2004; Xie et al. 2008). LKB1 can be phosphorylated on several residues, and recent evidence suggests that phosphorylation of LKB1 at Ser428 increases export of LKB1 from the nucleus and influences the ability of LKB1 to bind and phosphorylate AMPK at Thr172 (Song et al. 2008; Xie et al. 2006, 2008). In addition, LKB1 itself has roles in regulating cellular polarity and structure, such as promoting axon initiation during neuronal polarization in cultured hippocampal neurons (Shelly et al. 2007) and regulating neuronal migration and neuronal differentiation in the developing neocortex (Asada et al. 2007).

Neurite outgrowth is an early process of neuronal differentiation, which can be regulated by a large number of signals, such as mitogen-activated protein kinase and glycogen synthase kinase pathways (Chen et al. 2009; DeFuria and Shea 2007). Considering the potential role of AMPK in neuronal development, we hypothesized that impairment of AMPK activation was involved in the neurodevelopmental toxicity induced by As. Using the Neuro-2a (N2a) neuronal cell model, we designed the present study to determine whether As induced neurodevelopmental toxicity and to investigate the role of AMPK in this process. N2a, a mouse neuroblastoma cell line, is widely used to study the mechanisms of neuronal differentiation. In response to serum starvation or treatment with retinoic acid (RA) or dibutyryl cyclic AMP, N2a cells undergo neuronal differentiation characterized by cell cycle arrest, neurite outgrowth, and up-regulation of neurofilament (NF) proteins (Dasgupta and Milbrandt 2007). In the present study, we determined that As inhibited neurite outgrowth in N2a cells; this inhibitory effect was induced by suppressing the LKB1–AMPK pathway, which appears to play an important role in neuronal differentiation. Antioxidants, such as N-acetyl cysteine (NAC) and superoxide dismutase (SOD), antagonized the inhibitory effect of As, indicating that reactive oxygen species (ROS) play an important role in neuronal differentiation and mediating the action of As.

## Materials and Methods

### Chemicals

Nucleofector Kit V was purchased from Amaxa (Cologne, Germany), protein G-Sepharose beads were purchased from Amersham Biosciences (Pittsburgh, PA, USA), and other chemicals were obtained from Sigma Chemical Co. (St. Louis, MO, USA) unless otherwise stated. We purchased STRAD and lamin A/C antibodies from Santa Cruz Biotechnology Inc. (Santa Cruz, CA, USA) and other antibodies from Cell Signaling Technology Inc. (Beverly, MA, USA). Plasmids encoding a c-*myc*–tagged constitutively active form of *AMPK*α (*CA-AMPK*) or a dominant negative form of *AMPK*α (*DN-AMPK*) were generous gifts from J. Suttles (Louisville, KY, USA).

### Cell cultures and N2a cell differentiation

N2a cells were cultured in Dulbecco’s modified Eagle’s medium (DMEM) supplemented with 10% fetal bovine serum (FBS), 2 mM l-glutamine, 100 U/mL penicillin, and 100 μg/mL streptomycin at 37°C in 5% CO_2_ in a humidified atmosphere. To induce differentiation, growth medium was carefully removed and then replaced with an equal volume of DMEM supplemented with 2% FBS, 2 mM l-glutamine, 100 U/mL penicillin, 100 μg/mL streptomycin, and 20 μM all-*trans* RA. Arsenic trioxide was dissolved in 1 N sodium hydroxide and then diluted to 1 mM with phosphate-buffered saline (PBS); this was used as stock solution.

### Cytotoxicity assessment

Confirmation of cell viability was performed and quantified by the 3-(4,5-dimethyl-thiazol-2-yl)-2,5-diphenyltetrazolium bromide (MTT) assay as previously described (Wang et al. 2007).

### Quantification of neurite outgrowth

To count the number of cells expressing neurites and measure neurite length, we stained cells using crystal violet. Briefly, cultures differentiated in the presence or absence of As in six-well plates were washed in PBS before fixation with ice-cold methanol at –20°C for 15 min; cells were then stained with 0.5% crystal violet solution in methanol for 30 min at room temperature. Using an inverted light microscope at 320× magnification, we scored for the percentage of cells expressing neurites and determined average neurite length. Cells with neurites were defined as cellular extensions greater than two cell body diameters in length (Keilbaugh et al. 1991). Neurite length was measured as the distance from the center of the cell soma to the tip of its longest neurite (Chen et al. 2009). Five random fields were examined from each well, giving a total cell count of at least 200 cells/well. Each data point represents the mean of three individual wells in one experiment, and each experiment was repeated three times.

### Immunoblotting and immunoprecipitation

Fractionation of cytoplasm and nuclear protein was achieved as previously described (Wang et al. 2007). Briefly, N2a cells were lysed in an ice-cold lysis buffer [5 mM EDTA, 1% NP-40 (nonyl phenoxylpolyethoxylethanol), 10 mg/mL phenylmethylsulfonyl fluoride, 10 μg/mL leupeptin, and 100 mM sodium orthovanadate in PBS] and centrifuged at 20,800 × *g* for 10 min. The supernatant was designated as the cytoplasmic fraction. The pellets were sonicated in a nuclear extraction buffer [20 mM Tris-HCl, pH 7.5, 1% sodium dodecyl sulfate, 5 mM EDTA, 0.5% Triton X-100, 150 mM NaCl, 1 mM dithiothreitol, 10 μg/mL leupeptin, and 1 mM 4-(2-aminoethyl) benzenesulfonyl fluoride hydrochloride] and centrifuged at 20,800 × g for 10 min. The supernatant was collected and designated as the nuclear fraction.

The procedure for immunoblotting and immunoprecipitation has been previously described (Wang et al. 2007). Each experiment was repeated three times independently. The signal was analyzed by quantitative densitometry using ImageJ software (version 1.42; National Institutes of Health, Bethesda, MD, USA).

### Immunofluorescence

Immunocytofluorescent staining of phosphorylated LKB1(Ser428) [p-LKB1(Ser428)] was performed as previously described (Wang et al. 2007). N2a cells cultured on coverslips were treated with RA in the absence or presence of As for 24 hr and then fixed with 4% paraformaldehyde (15 min at room temperature). After incubation with the primary antibody (1:500) overnight at 4°C, p-LKB1(Ser428) in N2a cells was located using an antibody conjugated to Alexa-488. Nuclei were labeled with 4,6-diamindino-2-phenylindole (DAPI; 1 μg/mL in PBS). Images of fluorescence were acquired using the Leica TCS SP confocal laser-scanning microscope (Leica, Heidelberg, Germany).

### Cell transfection

N2a cells were cultured for 2 days before transfections. According to the manufacturer’s protocol, cells were transfected with either CA-AMPK or DN-AMPK plasmid using a Nucleofector instrument (Amaxa) and Nucleofector Kit V optimized for use with N2a cells. Briefly, 2 × 10^6^ cells were resuspended in 100 μL transfection buffer, and DNA plasmid was added to cells that were transferred to the cuvettes and electroporated using program T-24 (Amaxa).

### ROS measurement

We detected ROS using the fluorescent dye dichlorodihydrofluorescein acetate (DCFDA) and the hydroethidine (HE) staining method (Liao et al. 2000). HE is selectively oxidized by the superoxide anion (O_2_·^−^) into fluorescent ethidium, and DCFDA labels oxidation by hydrogen peroxide (H_2_O_2_), peroxynitrite, or the hydroxyl radical into fluorescent dichlorodihydrofluorescein (DCF). After treatment with RA in the presence or absence of As, N2a cells were collected, washed, incubated with 10 μM DCFDA or 5 μM HE for 30 min at 37°C, and then analyzed with a FACSCalibur flow cytometer (Becton Dickinson, Franklin Lakes, NJ, USA).

### Statistical analysis

Differences among treatment groups were tested using analysis of variance. We consider a *p*-value of < 0.05 statistically significant. In cases where significant differences were detected, we performed specific post hoc comparisons between treatment groups using Student-Newman-Keuls tests. The analyses were performed using SPSS software (version 10.0; SPSS Inc., Chicago, IL, USA).

## Results

### Arsenic inhibits neurite outgrowth

**Figure 1:**
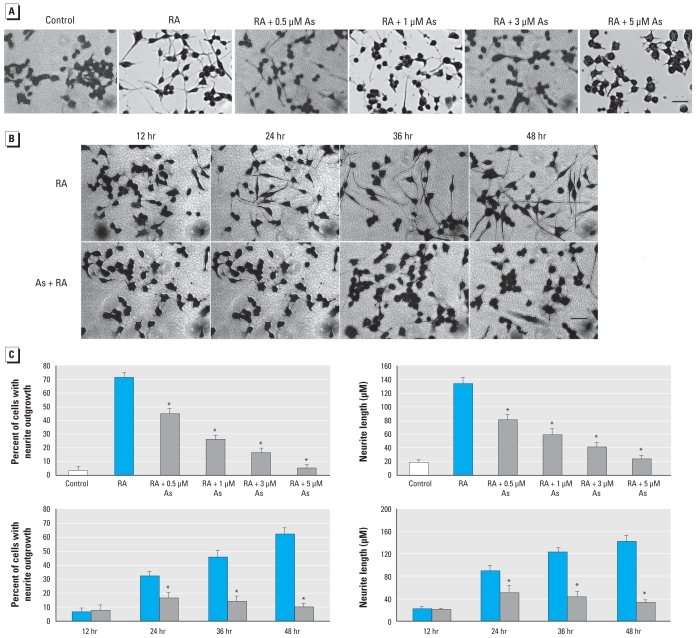
Arsenic inhibits neurite outgrowth in N2a cells. Cells were induced to differentiate for 48 hr in media containing RA or RA plus 0.5–5 μM As (*A*) or for 0–48 hr in growth medium alone without treatment (control), RA, or RA plus 3 μM As (*B*). Images are typical fields of cells viewed with an inverted light microscope; bars = 100 μm. (*C*) Percentage of cells exhibiting neurites (left) and the average length of neurites (right) in 200 cells treated as in *A* (top) and as in *B* (bottom). Each data point is the mean ± SE of three independent experiments. *p <0.05 compared with RA treatment alone.

To determine the effect of As exposure on neuronal differentiation, we treated differentiating N2a cells with various concentrations of As (0–10 μM) for 0–48 hr. As shown in Figure 1A, RA treatment resulted in visible changes in cell morphology, such as shrinkage of the cell body and neurite outgrowth (appearance of processes longer than the cell body). Combined RA/As treatment exerted an inhibitory effect on neurite outgrowth in a dose-dependent (Figure 1A) and time-dependent (Figure 1B) manner. We observed a noticeable reduction in both the number of cells bearing neurites and neurite length, compared with RA treatment alone (Figure 1A, B). The number of neurite-bearing cells and the length of neurites were reduced by 55% and 69%, respectively, after exposure to 3 μM As for 48 hr (Figure 1C). To determine whether this effect was a consequence of general toxicity due to As, we measured cell viability by the MTT assay. Arsenic concentrations of 0.5–5 μM had no effect on the cell viability, whereas 10 μM As caused 35% cell death after 48 hr exposure [see Supplemental Material, Figure 1 (doi:10.1289/ehp.0901510)]. Based on these results, we selected 3 μM As for all of our following studies. NFs and microtubule-associated protein-2 (MAP2) are critical for neurite outgrowth and dendrite development. Consistent with morphological differentiation, NFs and MAP2 were down-regulated by As exposure. Immunocytochemistry with antibody against the neuron-specific protein MAP2 revealed reduced neurite outgrowth in cells exposed to As (See Supplemental Material, Figure 2). These data suggest that As at low exposure levels inhibited neurite outgrowth in N2a cells.

### Arsenic inhibits activation of AMPK

**Figure 2:**
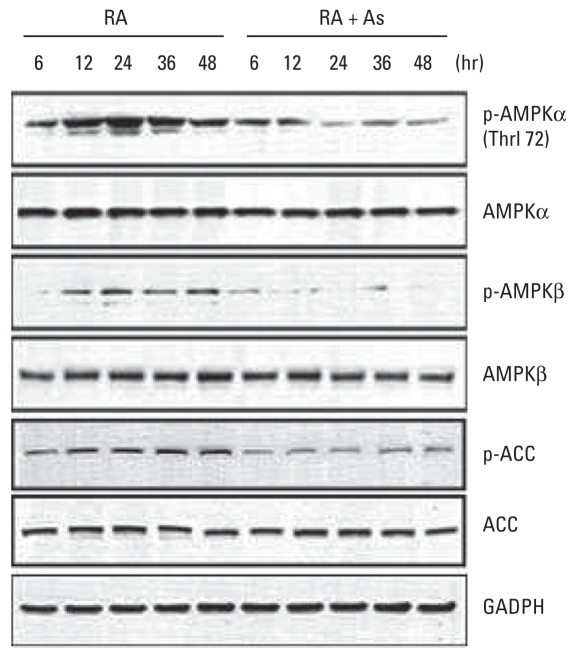
Arsenic inhibits AMPK activation in N2a cells treated with RA or RA plus 3 μM As for 6–48 hr. The expression of AMPK, ACC, p-AMPK, and p-ACC was determined with immunoblotting. GADPH was used as the loading control in the immunoblot.

AMPK activation requires phosphorylation of Thr172 in the activation loop of its catalytic α subunits [AMPK(Thr172)]. We sought to determine whether As affected the activation of AMPK in differentiating N2a cells. As shown in Figure 2, RA treatment resulted in a robust increase in phosphorylated AMPK(Thr172) [p-AMPKα(Thr172)] within 6 hr that persisted for up to 48 hr. Exposure to 3 μM As for 24 hr resulted in rapid and continuous reduction in AMPKα phosphorylation, decreasing by 90% compared with RA treatment. This was also accompanied by a rapid and sustained decrease in phosphorylation of the AMPK β subunit (AMPKβ), a positive regulator of AMPK activity [see Supplemental Material, Figure 3 (doi:10.1289/ehp.0901510)]. To confirm the effect of As on AMPK activation, we monitored phosphorylation of acetyl-coenzyme A carboxylase (ACC), a primary target of activated AMPK. The profile of As-induced decrease of phosphorylated ACC (p-ACC) was similar to that of p-AMPKα(Thr172). Total AMPK and ACC levels changed minimally at all the time points in both RA- and RA/As-treated groups.

### Arsenic inhibits neurite outgrowth by blocking AMPK activation

**Figure 3:**
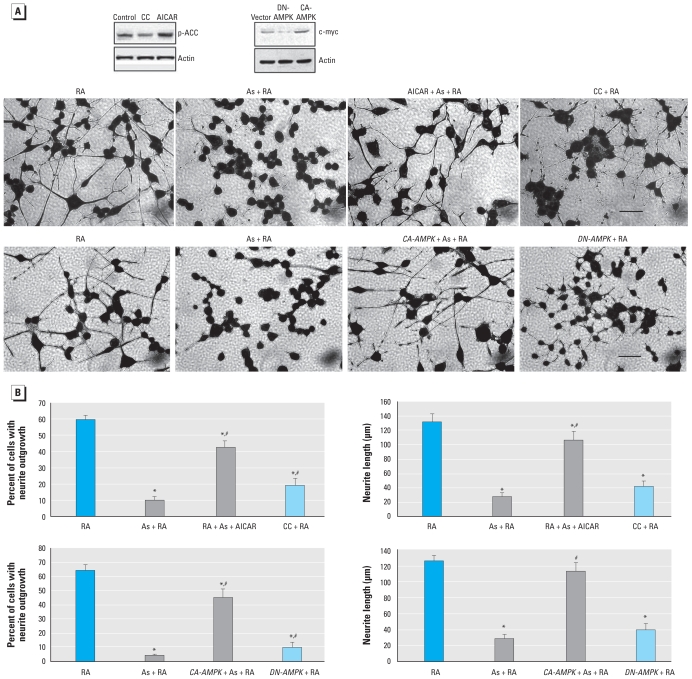
AMPK inactivation causes As-induced inhibition of neurite outgrowth. (*A*) Effect of pretreatment with 4 mM AICAR or 20 μM CC (or transfection with CA-AMPK or DN-AMPK on neurite outgrowth in differentiating N2a cells exposed to RA or RA plus 3 μM As for 48 hr. Blots show the efficiency of CC, ACAR, DN-AMPK, and CA-AMPK on the activation of AMPK using the expression of p-ACC and c-*myc*. Images represent typical fields of cells viewed with an inverted light microscope; bars = 100 μm. (*B*) Percentage of cells exhibiting neurites (left) and average neurite length (right) in 200 cells. Each data point is the mean ± SE of three independent experiments. *p **<**0.05 compared with RA treatment alone. ^#^*p* < 0.05 compared with RA plus As-treatment.

To verify the role of AMPK in As-induced inhibition on neurite outgrowth, we used an AMPK pharmacological activator or genetic manipulation with a CA-AMPK plasmid. Pretreatment with 4 mM 5-aminoimidazole-4-carboxamide riboside (AICAR) or transfection with CA-AMPK protected differentiating N2a cells against inhibition of neurite outgrowth induced by As (Figure 3A), by increasing the percentage of cells bearing neurites and restoring the average neurite length to values comparable with those from RA treatment (Figure 3B). Even after reestablishing AMPK activity, neurite outgrowth continued to be lower in the As-treated cells than in those treated only with RA (Figure 3B). Other targets of LKB1 could be acting in parallel, which could be less sensitive to As. Pretreatment with 20 μM compound C (CC), an AMPK inhibitor, or expression of *DN-AMPK* in RA treatment culture had an inhibitory effect on neurite outgrowth similar to that of As (Figure 3A, B), indicating the important role of AMPK during neurite differentiation. Taken together, these results indicate that AMPK inactivation plays an important role in As-induced inhibition of neurite outgrowth.

### Arsenic inhibits activation of LKB1

**Figure 4:**
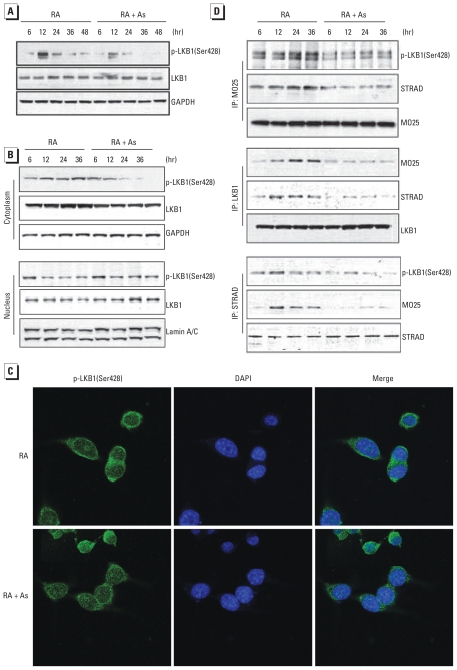
Arsenic inhibits LKB1 activation. N2a cells were treated with RA or RA plus 3 μM As. Immunoblots showing expression of LKB1 and p-LKB1(Ser428) (*A*) and effect of As on p-LKB1(Ser428) distribution (*B*) in N2a cells at five time points; GADPH and lamin A/C served as loading controls of cytoplasm and nucleus, respectively. (*C*) Effect of As on localization of p-LKB1(Ser428) in N2a cells treated for 24 hr. Immunofluorescence indicates expression of p-LKB1(Ser428) as green, and nuclei (blue) were visualized with DAPI staining. (*D*) Effect of As on LKB1/MO25/STRAD interaction in treated N2a cells at four time points. IP, immunoprecipitated.

To evaluate the upstream kinase affected by As in blunted AMPK activation, we examined the effect of As on the activation of LKB1, a major AMPK-kinase, in N2a cells. Phosphorylation of LKB1(Ser428) induced by RA was decreased by 56% after 12 hr of exposure to As and remained at a very low level until 48 hr [Figure 4A; see also Supplemental Material, Figure 4 (doi:10.1289/ehp.0901510)]. Overall LKB1 expression was not changed by As exposure. However, the distribution and expression of p-LKB1(Ser428) in the cytoplasm differed between RA and combined RA/As treatment (Figure 4B; see Supplemental Material, Figure 5). RA treatment increased the level of p-LKB1(Ser428) during the early stage of differentiation, which remained at high levels until 36 hr, whereas As exposure inhibited the increase. In the nuclear fraction, the RA-induced decrease in p-LKB1(Ser428) was blocked by As. In the absence of As, the profile of expression of LKB1 in cytoplasm and nucleus was similar with that of p-LKB1(Ser428) in cytoplasm and nucleus, respectively. Because overall LKB1 expression was not changed by As, this profile suggested that As blocked the RA-induced translocation of LKB1 from nucleus to cytoplasm in differentiating N2a cells.

We further examined the subcellular distribution of p-LKB1(Ser428) in N2a cells with immunofluorescence staining. Consistent with the observation obtained from immunoblotting analysis, As failed to stimulate and induce cytoplasm translocation of p-LKB1(Ser428) (Figure 4C). To confirm the inactivation of LKB1, we examined the effect of As on LKB1/STRAD/MO25 interaction in N2a cells with a coimmunoprecipitation assay. RA treatment increased the association of LKB1, MO25, and STRAD during the period of neurite outgrowth (Figure 4D); combined RA/As treatment decreased that association but did not affect their expression. Taken together, these results indicate that LKB1 inactivation plays a role in inhibition of AMPK activation by As.

### ROS suppress the LKB1–AMPK pathway

**Figure 5:**
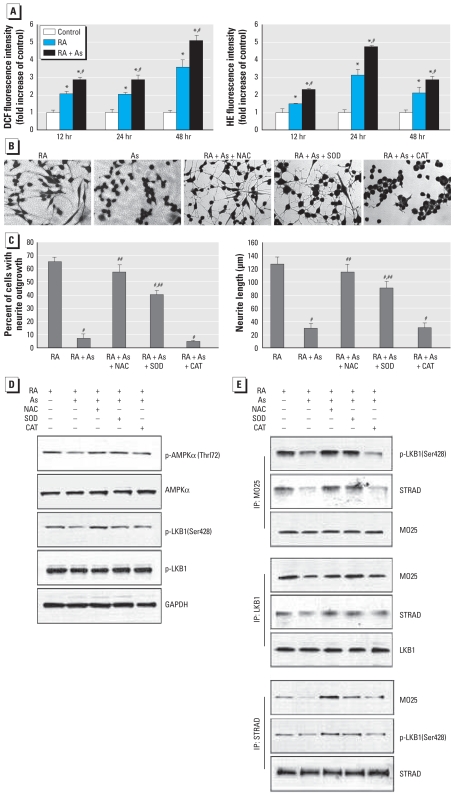
The role of ROS on the reduced neurite outgrowth in N2a cells exposed to RA or RA plus 3 μM As. Abbreviations: +, with; −, without; IP, immunoprecipitated. (*A*) ROS production evaluated by intensity of DCF and HE fluorescence (fold increase of control); each data point represents the mean ± SE of three independent experiments. (*B, C*) Effect of the antioxidants NAC, SOD, and catalase (CAT) on reduced neurite outgrowth induced by As. (*B*) Images of typical fields of cells viewed with an inverted light microscope; bar = 100 μm. (*C*) Percentage of cells exhibiting neurites (left) and average neurite length (right) in 200 cells; each data point represents the mean ± SE of three independent experiments. (*D*) Effect of antioxidants on the expression of p-AMPKα(Thr172) and p-LKB1(Ser428) in differentiating cells exposed to As for 24 hr; GADPH served as the loading control. (*E*) Effect of antioxidants on association of LKB1/MO25/STRAD in differentiating cells exposed to As for 24 hr. *p < 0.05 compared with control. #*p* < 0.05 compared with RA treatment. ^##^*p* < 0.05 compared with RA plus As treatment.

Arsenic is a well-known ROS inducer, and generation of ROS associated with As exposure has been shown to play a fundamental role in the induction of adverse health effects (Shi et al. 2004). The role of ROS in reduced neurite outgrowth has been explained by the observation that ROS induces reconfiguration of microtubules (He et al. 2002; Ibi et al. 2006). We therefore sought to determine whether oxidative stress was involved in the reduced neurite outgrowth induced by As. The fluorescence intensity produced by both DCFDA and HE was significantly higher in differentiating cells than in untreated control cells (Figure 5A). Combined RA/As treatment stimulated greater formation of ROS compared with RA treatment. We observed the higher fluorescence levels from 6 hr in both RA and combined RA/As treatment (data not shown), and these higher levels persisted for up to 48 hr. The intensity of HE reached a maximum increase at 24 hr with both RA and combined RA/As treatment, whereas the intensity of DCF peaked at 48 hr. These results indicate that more ROS was produced by As during neurite outgrowth.

To verify the role of ROS in reduced neurite outgrowth induced by As, we examined the effects of the antioxidants NAC (a potent antioxidant), SOD (an O_2_·^−^ scavenger), and catalase (an H_2_O_2_ scavenger) (Figure 5B, C). When 2 mM NAC or 500 U/mL SOD was included in the medium with RA plus As for 48 hr, we observed that the inhibitory effect of As on neurite outgrowth was significantly reversed, as shown by increased percentages of cells bearing neurites and greater neurite elongation (Figure 5C). NAC completely reversed the reduction in neurite outgrowth induced by As. In contrast, inclusion of 500 U/mL catalase did not affect neurite outgrowth (Figure 5C). We observed no significant change in cell viability in the presence of these reagents (data not shown). Accordingly ROS, especially O_2_·^−^, but not H_2_O_2_, appears to be involved in the reduced neurite outgrowth induced by As. To identify the effect of ROS on the LKB1–AMPK pathway, we examined the effects of the antioxidants NAC, SOD, and catalase on phosphorylation levels of AMPK and LKB1. As shown in Figure 5D, pretreatment with NAC or SOD, but not catalase, protected against the As-induced decrease in p-AMPK(Thr172) and p-LKB1(Ser428) and had no effect on protein expression. We further examined the effect of ROS on the association of LKB1, MO25, and STRAD and found that the disrupted association of LKB1, MO25, and STRAD induced by As was reversed by pretreatment with NAC or SOD but not catalase (Figure 5E). Taken together, these results verify that ROS, especially O_2_·^−^, plays a critical role in reduced neurite outgrowth induced by As through the LKB1–AMPK pathway.

## Discussion

In the present study we found that As inhibits neurite outgrowth in N2a cells, and this inhibition is due to deficient activation of AMPK as a consequence of decreased activation of LKB1. Arsenic suppresses LKB1 activity and the translocation from nucleus to cytoplasm by disrupting the association of the LKB1/MO25/STRAD complex and inhibiting the phosphorylation of LKB1(Ser428). The antioxidants NAC and SOD antagonize the inhibitory effect of As by reversing decreased p-AMPK(Thr172) and p-LKB1(Ser428) levels and restoring disrupted LKB1/MO25/STRAD association. The results suggest that ROS plays an important role in neuronal differentiation and mediating the action of As.

Arsenic at low concentrations (≤ 5 μM) inhibits neurite outgrowth, as demonstrated by decreased percentages of cells bearing neurites and decreased neurite length, which is consistent with other As studies (DeFuria and Shea 2007; Frankel et al. 2009). Arsenic, at concentrations of 5 and 10 μM, disrupts neurite growth and complexity in differentiating PC12 pheochromocytoma cells (Frankel et al. 2009). Arsenic concentrations < 1 μM inhibit NF transport into axons in NB2/d1 cells and cultured dorsal root ganglion neurons (DeFuria and Shea 2007). However, some studies have reported that low As concentrations (0.5–1 μM) stimulate neurite outgrowth (Jung et al. 2006). One explanation for the observed differences in results is the differing cell culture conditions used during the differentiation treatments, most notably the presence or absence of serum. However, this is not likely to be the only contributing factor.

Arsenic-induced inhibition of neurite outgrowth is mediated by its modulation of AMPK activity, which is crucial for maintaining the structural and functional integrity of neurons, as demonstrated by the loss of axonal and dendritic processes in β- (Spasić et al. 2008) or γ- (Tschäpe et al. 2002) subunit–deficient *Drosophila* models, and for contributing to robust neurite outgrowth in N2a cells induced by resveratrol (Dasgupta and Milbrandt 2007). Compared with the RA treatment, combined RA/As treatment inhibited phosphorylation of AMPKα(Thr172), as well as phosphorylation of ACC, a substrate of AMPK. The phosphorylation of AMPKβ, which mediates the association of the AMPK heterotrimeric complex, also decreased in a time-dependent manner. Pretreatment with the AMPK pharmacological activator AICAR or transfection with a CA-AMPK plasmid led to a recovery effect on reduced neurite outgrowth induced by As, suggesting that blunted AMPK activation is responsible for the inhibitory effect of As.

The loss of AMPK activity induced by As is due to the suppression of LKB1 activity and translocation, by disturbing the association of the LKB1/MO25/STRAD complex, as well as inhibiting the phosphorylation of LKB1(Ser428). AMPK activity is regulated through phosphorylation at Thr172 by the upstream serine/threonine kinase LKB1, the localization of which depends on its kinase activity. LKB1 forms a heterotrimeric complex with STRAD and MO25, which are required for its activation and cytosolic localization. In the absence of these proteins, LKB1 is localized to the nucleus. Formation of the LKB1/MO25/STRAD complex causes a relocalization of LKB1 to the cytosol and enhances LKB1 activity (Boudeau et al. 2003; Shaw et al. 2004). Phosphorylation of LKB1(Ser428) is reported to result in translocation of LKB1 and increase association of LKB1 with AMPK (Xie et al. 2006). In addition, interaction of STRAD with LKB1 promotes phosphorylation of LKB1 (Shelly et al. 2007). The present study demonstrates that As time-dependently decreases p-LKB1(Ser428) and disrupts LKB1/STRAD/MO25 association, which blocks cytoplasm translocation of LKB1. The inhibitory effect of As on LKB1 translocation from nucleus to cytoplasm suggests that LKB1 inactivation is involved in deficient activity of AMPK induced by As. Besides LKB1 activation, AMPK can also be activated by CaMKKβ, which is stimulated by intracellular calcium (Carling et al. 2008). Several recent studies have reported that As increases the intracellular calcium overload, and the increase is not reversible if As is removed (e.g., Günes et al. 2009). In the present study we found no obvious difference in the expression of CaMKKβ between RA and treatment with RA plus As [see Supplemental Material, Figure 6 (doi:10.1289/ehp.0901510)]. Thus, the loss of AMPK activity induced by As is likely caused by LKB1 inactivation.

The present study also shows that oxidative stress induced by As, especially O_2_·^−^, plays a critical role in blocking the LKB1–AMPK pathway. Modulation of ROS levels influences multiple aspects of neuronal differentiation and function (Tsatmail et al. 2006). Physiological levels of ROS are critical for maintaining a dynamic F-actin cytoskeleton and controlling neurite outgrowth (Munnamalai and Suter 2009). However, excessive ROS plays a neurotoxic role during differentiation that likely contributes to brain dysfunction (Lee et al. 2002; Tsatmail et al. 2006). In our study, the antioxidants NAC and SOD protected against reduced neurite outgrowth induced by As by recovering As-disrupted LKB1/MO25/STRAD association and the level of p-LKB1(Ser428). The function of SOD is to catalyze the dismutation reaction of O_2_·^−^ to generate H_2_O_2_. Our results indicate that H_2_O_2_ may not play an important developmental neurotoxic role in the As model, because its formation should have been greatly increased by the supplemented SOD. Instead, an increase in the steady-state levels of O_2_·^−^ appears to be pivotal to inhibiting neurite outgrowth. An important issue for understanding the physiological level of ROS in neuronal differentiation concerns the intriguing role of ROS during differentiation. High ROS levels have been reported to be transient *in vivo* during neuronal development, suggesting that some feedback loop must exist to decrease the levels of ROS during neuron differentiation (Tsatmail et al. 2006). Mutation in the human copper/zinc SOD gene results in defects in neurite outgrowth, leading to the decrease in the amount of NFs and MAP2 (Lee et al. 2002). Resveratrol promotes development by significantly increasing manganese SOD expression and intracellular glutathione level (Kao et al. 2010). Taken together, excess ROS induced by As may play a critical role in inhibiting neurite outgrowth and the LKB1–AMPK pathway.

## Conclusion

Results of the present study demonstrate that reduced neurite outgrowth induced by As results from inhibition of AMPK activity as a consequence of LKB1 inactivation. Arsenic suppresses LKB1 activity and translocation from nucleus to cytoplasm by disrupting the association of the LKB1/MO25/STRAD complex, as well as inhibiting the phosphorylation of LKB1(Ser428). ROS induced by As, especially excessive O_2_·^−^, plays a critical role in blocking the LKB1–AMPK pathway. Our studies provide insight into the underlying mechanisms of As-induced damage to the developing CNS and provide important information for therapeutic approaches.
